# Beyond words: an investigation of fine motor skills and the verbal communication spectrum in autism

**DOI:** 10.3389/fpsyt.2024.1379307

**Published:** 2024-05-21

**Authors:** Marian Simarro Gonzalez, Gessica Ni, Valerie Lam, Carly Demopoulos

**Affiliations:** ^1^Spoken Language Interest Group, Basque Centre on Cognition, Brain and Language, San Sebastian, Spain; ^2^Department of Psychiatry and Behavioral Sciences, University of California, San Francisco, San Francisco, CA, United States; ^3^Department of UCSF Epilepsy and Pediatric Brain Center, University of California, San Francisco (UCSF) School of Medicine, San Francisco, CA, United States; ^4^Department of Neurology and Pediatrics, University of California, San Francisco, San Francisco, CA, United States; ^5^Department of Radiology and Biomedical Imaging, University of California, San Francisco, San Francisco, CA, United States

**Keywords:** autism, motor skills, speech, language, communication

## Abstract

**Introduction:**

This study investigated the associations between fine motor skills and expressive verbal abilities in a group of 97 autistic participants (age 8-17, mean=12.41) and 46 typically developing youth (age 8-17, mean=12.48).

**Methods:**

Participants completed assessments of motor and verbal communication skills, including finger tapping speed, grooved pegboard, grip strength, visual-motor integration tasks, and measures of speech and communication skills. ASD group performance on motor tests was compared to controls. Non-parametric tests were used to analyze group differences and correlations between motor and verbal communication skills. Based on prior research, we hypothesized that individuals on the autism spectrum would exhibit deficits in fine motor speed, dexterity, pencil motor control, but not manual motor strength. Additionally, we expected that impaired fine motor skills would be linked to poorer performance on standardized measures of verbal abilities.

**Results:**

The results indicated that 80% of autistic participants demonstrated an impairment on at least one measure of motor skills, and as a group, they exhibited significantly poorer fine motor performance compared to the non-ASD group in dominant hand finger tapping speed, bilateral fine motor dexterity measured via the grooved pegboard task, and pencil motor coordination and visual-motor integration measured on the Beery-Buktenica Developmental Test of Visual-Motor Integration-Sixth Edition. Moreover, impaired fine motor skills were associated with poorer performance on standardized clinical measures of verbal abilities, including articulation errors, receptive and expressive language and vocabulary, rapid naming, oromotor sequencing, and parent reported functional communication skills and social communication symptoms.

**Discussion:**

Overall,our findings suggest there is a high prevalence of fine motor impairments in ASD, and these impairments were associated with a range of verbal abilities. Further research is warranted to better understand the underlying mechanisms of these associations and develop targeted interventions to address both fine motor and verbal impairments in ASD.

## 1 Introduction

Children’s language, cognition, and social skills development are closely linked to their motor function development (1, 2). Previous research has suggested that motor impairments in infants at risk for, or diagnosed with, autism spectrum disorder (ASD) appear before more salient social-communicative impairments are formally diagnosed (3, 4). The expanding body of research on motor development in ASD suggests that autistic children often face continued challenges with gross and fine motor abilities (5, 6), and these motor differences have been associated with other core ASD symptomatology, including language (7–9) and social skills (10, 11). Despite recognizing motor delays in ASD, specifics about the affected motor skills remain unclear (12–14). Investigating motor abilities and their association with language development during childhood through adolescence in autistic individuals may yield valuable insights into the complex factors shaping their overall development. Such insights can inform targeted interventions aimed at addressing motor impairments in this population (15). Furthermore, early detection and intervention for motor difficulties may also lead to improvements in social communication for autistic individuals (16). Prior research on motor skills of autistic individuals has shown significant variation in research design, including the specific motor skills assessed (e.g., gait (17, 18), balance (19), ball skills (20), postural development (rolling, sitting, standing) (21) and object manipulation (8)). Measurement methods also vary considerably, including parental questionnaires (22, 23), standardized assessments (8, 24), behavioral coding of videos (25), and kinematic motion capture (18). Studies differ in participant characteristics as well, with research including children (21), adults (17), and individuals across the entire autism spectrum (14, 26, 27). Statistical control for confounding variables is also inconsistently applied, with some studies controlling for factors like IQ (25) while others do not (22). This heterogeneity makes it difficult to draw precise conclusions about which motor skills are impaired in ASD, the magnitude of impairments for different types of motor skills, the developmental trajectory of these impairments, whether motor skill deficits are independent of broader cognitive or developmental functioning, or whether motor skill deficits are associated with specific subgroups or features of autistic children (11).

Recent studies revealed variability in motor skills of autistic individuals based on parent-reported measures (5), standardized clinical assessments (6, 11), and neuroimaging techniques (28). Specifically, autistic children had lower scores on parent-report measures of motor skills compared to neurotypical children when measured via the Childhood Autism Rating Scale: Motor (CARS-M; 29), the Developmental Coordination Disorder Questionnaire (DCDQ; 30), and the Movement Assessment Battery for Children (MABC-2; 23, 31). Parent report measures can be quickly administered without requiring the child’s active participation. This is particularly advantageous for autistic children who may face challenges in traditional testing scenarios (32). Additionally, these measures offer insights into motor skills across diverse settings, including home, school, and social environments, aiding in the identification of strengths and weaknesses in motor development (33). However, such measures come with some limitations, such as the subjective nature of parent observations, potential inaccuracies due to lack of training, and the focus on observed behavior rather than the child’s full capability.

For this reason, performance-based standardized clinical assessments can be used to add additional information about a person’s abilities by testing their performance in relation to age-matched peers. Many studies have reported delays and impairments in gross motor skills in autism. A systematic review and meta-analysis by Whyatt & Craig (23) reported that manual dexterity and ball skills (i.e., the ability to accurately and efficiently throw, strike, catch, and kick objects) were more impaired than other motor skills, including balance, in autistic individuals, and that object control skills were the only motor skills that predicted later ASD symptom severity. The authors suggested that these impairments may be due to deficits in perception-action coupling, the ability to use sensory information to guide movement. These findings suggest that object control skills may be particularly important for understanding the fundamental mechanisms underlying motor impairment in autism. This is because object control skills require continuous in-the-moment integration of sensorimotor feedback to adjust motor output. In a large sample (N=1094), Gandotra et al. (34) identified a high prevalence (63–82%) of fundamental movement skill (FMS; i.e., balance, object control, and locomotor skills) impairments in a systematic review among autistic individuals, who on average performed 6.4 months behind age expectations. The difference between chronological age and FMS age equivalent increased progressively with age. In addition, children diagnosed with ASD exhibited greater deficits in FMS competencies, particularly in object control (53–82%), locomotor skills (67–80%), and balance skills (33–58%) when compared to neurotypical children. Furthermore, studies including participants with greater support needs reported a higher prevalence of FMS impairment (35). However, due to gender imbalance in the aforementioned studies, with 85% of participants being male, the results may not be generalizable to autistic females.

These motor difficulties extend beyond gross motor skills and encompass fine motor skills as well. Studies examining both gross and fine motor functioning have reported difficulties in activities like running and jumping, as well as visual motor integration tasks, such as eye-hand coordination and tracing (36, 37). The differences in motor skills between children with and without autism were found to be most pronounced in fine motor skills, such as hand coordination and dexterity (6). Infants with high‐chance of ASD based on family history have been shown to have less developed object manipulation on the Mullens Scale of Early Learning (MSEL, 38) and weaker fine motor and decreased grasping activity compared to infants with no familial history (low-chance) of ASD (37). Notably, both groups performed in the typical range on average, despite the lower scores in the ASD group. Longitudinal assessments indicate that grasping activities in high-chance infants increased between 6 and 10 months of age to a level comparable to that displayed by same-age peers in the low-chance group (37). This suggests that high-chance infants may be able to catch up to their peers in terms of motor development, and that motor skills may be delayed rather than impaired.

In contrast, other research suggests that motor impairments persist over time in autistic children compared to their neurotypical counterparts and that the gross motor delays were more prominent in older children who were 37–60 months old (6). Similarly, fine motor delays in children with ASD have been shown to increase over time. For example, toddlers on the autism spectrum between the ages of 12 and 36 months had significantly lower scores on the MSEL motor scale than typically developing toddlers, and these disparities in gross and fine motor abilities became more pronounced with each 6-month period of chronological age (39). Landa and Garrett-Mayer (40), in their prospective study of 87 infants at risk for ASD using MSEL, found that the children in the ASD group performed significantly worse than the other groups in their gross and fine motor skills as early as 14 months and nearly half of the ASD group showed ‘developmental worsening’ between 14 and 24 months. These studies indicate that delays and/or atypical patterns in fine motor development appear to emerge early in autistic children and persist over time. For instance, Messing & Apthorp (27) examined upper limb motor skills in adolescents with ASD (aged 12–17 years) and found persistent difficulties with motor coordination. Similarly, Duffield et al. (26) focusing on fundamental motor skills in children (aged 7–12 years) with ASD reported impairments in balance and agility and found that adolescents with ASD exhibited poorer balance compared to typically developing peers, and Faber et al. (14) investigating gait patterns in autistic youth (aged 10–18 years) identified atypical gait characteristics. Motor skills development plays a crucial role in childhood, impacting social participation, independence, and overall well-being. However, even though up to 87% of individuals with autism often exhibit challenges in motor skills that convey early developmental risk and are clinically significant and treatable, these motor challenges yet are often overlooked and underrecognized (16).

The neurobiology of motor differences has also been examined in autism. Children with “high functioning autism” (HFA) have shown different patterns of cerebellar activity than neurotypical children during a finger tapping motor task and have reduced functional connectivity between the cerebellum and other brain regions involved in motor execution (28). Specifically, while both groups exhibited anticipated primary activations in cortical and subcortical regions associated with motor execution, the neurotypical group demonstrated greater activation in the ipsilateral anterior cerebellum whereas the high-functioning autism group displayed heightened activation in the supplementary motor area. In addition, the children with high-functioning autism showed reduced connectivity throughout the motor execution network compared to the control children.

In sum, the prior research on motor development in ASD suggests that motor delays are apparent early in development, often before symptoms of ASD manifest, and are associated with social communication differences. These motor delays may become more pronounced over time relative to neurotypical peers, although most studies using performance-based measures of motor skills have focused on young children. On the other hand, while there have been studies on the association between motor and language skills development, they are often in infants and toddlers (e.g. 41), but investigations into later childhood, adolescence, and young adulthood are crucial for understanding the persistent impact of motor skill challenges across the lifespan. This paper aims to contribute to a more comprehensive understanding of motor skills in ASD by focusing on autistic participants aged 8 to 17 years.

In the current study, we measured fine motor abilities of a group of autistic participants in late childhood through adolescence and examined associations between those motor skills and a wide range of verbal abilities. Based on prior research identifying fine motor differences in autism (12, 14, 26, 28, 42), we hypothesized that autistic participants will score lower on measures of fine motor speed, dexterity, and pencil motor control, but not manual motor strength (26). Based on early developmental evidence of associations between motor skills and language development (7–9), we also hypothesized that impaired motor skills would be associated with poorer performance on standardized clinical measures of verbal abilities.

## 2 Materials and methods

### 2.1 Participants

Participants were 97 English-speaking youth (59 males, 38 females) ages 8–17 (M = 12.41, SD = 2.7) with a DSM-5 (43) diagnosis of ASD and 46 non-autistic youth (22 males, 24 females), ages 8–17 (M = 12.48, SD = 2.52). Demographic data are presented in **Table 1**.

**Table 1:** Demographics (M ± SD [Range]).

	ASD Group	Control Group	Statistics (U)
Age	12.41 ± 2.7 [8-17.42]	12.48 ± 2.52 [8.42-16.83]	2202.50
Race (N [%])			
Caucasian	55 [56.7%]	21 [45.7%]	
Asian	14 [14.4%]	7 [15.2%]	
African American	1 [1%]	0	
Native American	1 [1%]	0	
Multiracial	24 [24.7%]	18 [39.1%]	
Other	2 [2.1%]	0	
Ethnicity (N [%])			
Hispanic	19 [19.6%]	7 [15.2%]	
Non-Hispanic	78 [80.4%]	39 [84.8%]	
Sex Assigned at Birth (N [%])			
Male	59 [60.82%]	22 [47.82%]	
Female	38 [39.18%]	24 [52.17%]	
Gender Identification (*N* [%])			
Female	32 [32.99%]	24 [52.17%]	
Male	59 [60.82%]	21 [45.65%]	
Nonbinary	3 [3.09%]	1 [2.17%]	
Transgender	3 [3.09%]	0	
Handedness (*N* [%])			
Left	12 [12.40%]	4 [8.70%]	
Right	80 [82.50%]	41 [89.10%]	
Ambidextrous	5 [5.20%]	1 [2.20%]	
WISC-V			
FSIQ	98.33 ± 23.21 [42-138]	115.63 ± 10.76 [85-129]	1051.50**
GAI	102.08 ± 23.14 [43-143]	117.33 ± 11.22 [86-133]	1224.00**
VCI	101.88 ± 25.97 [45-139]	115.76 ± 13.59 [81-150]	1445.50*
VSI	101.82 ± 20.99 [45-138]	114.52 ± 12.94 [89-141]	1362.50**
FRI	101.38 ± 19.69 [55-140]	113.7 ± 11.4 [91-140]	1279.00**
PSI	88.06 ± 19.9 [45-123]	104.02 ± 15.23 [69-155]	1135.50**
WMI	94.59 ± 22.98 [45-146]	107.15 ± 14.38 [79-142]	1414.50*
TONI-4	102.19 ± 14.55 [63-138]	108.02 ± 9.28 [88-126]	1607.50*
CELF-5			
ELI	97.13 ± 25.15 [45-139]	112.78 ± 12.62 [85-135]	1397.00**
RLI	96.39 ± 25.43 [45-141]	112.76 ± 10.26 [92-133]	1330.00**

* p < 0.01.

** p ≦ 0.001.

WISC-V, Wechsler Intelligence Scale-Fifth Edition; FSIQ, Full-Scale Intelligence Quotient; GAI, General Ability Index; FRI, Fluid Reasoning Index; PSI, Processing Speed Index; VCI, Verbal Comprehension Index; VSI, Visual Spatial Index; WMI, Working Memory Index; TONI-4, Test of Nonverbal Intelligence, Fourth Edition; CELF-5, Clinical Evaluation of Language Fundamentals-5^th^ Edition; ELI, Expressive Language Index; RLI, Receptive Language Index.

### 2.2 Procedures

After obtaining informed consent and assent, participants were scheduled for a diagnostic evaluation and neuropsychological testing over the course of 2–3 visits. Order of tests administered prioritized participant preferences and needs and thus was not standardized. Breaks and practice sessions were offered as needed, and when necessary, visits were broken up into shorter sessions to accommodate participant needs. All participants were administered the parent report measure, the Social Communication Questionnaire (SCQ; 44). ASD participants and those without a prior ASD diagnosis who scored >10 on the SCQ were evaluated for ASD using gold standard diagnostic tools, including the Autism Diagnostic Observation Schedule-2^nd^ Edition (ADOS-2; 45) and the Autism Diagnostic Interview-Revised (ADI-R; 46). Evaluation of symptoms according to DSM-5 diagnostic criteria was contextualized according to participants’ language and intellectual abilities, assessed on the Clinical Evaluation of Language Fundaments-5^th^ Edition (CELF-5; 47) and the Wechsler Intelligence Scale for Children-Fifth Edition (WISC-V; 48). The participants’ handedness was self-reported and confirmed via observation of hand selected for writing tasks. For participants whose handedness was described as ambidextrous, they were instructed to use their preferred hand first for motor tasks, and this hand was scored as the dominant hand for generation of norm-referenced scores.

### 2.3 Measures

#### 2.3.1 Motor tests

Finger tapping speed was assessed bilaterally via a board mounted tapper with counter (Finger Tapping Test; FTT; https://www.parinc.com/Products/Pkey/114) according to procedures outlined in the Halsted-Reitan Neuropsychological Battery (49). Specifically, participants used their index finger to depress and release the tapper to the point of advancing the counter. The number of taps completed in a 10 second window was recorded for each trial, separately for each hand. To ensure consistency of responses, a minimum of five trials was administered. If the total number of taps for each trial was not within 5 taps of all other trials, additional trials were administered until consistency was achieved. If consistency was not achieved within 10 trials, the number of taps was averaged across all 10 trials. This procedure was performed separately for each hand. Age scaled scores were then computed separately for the dominant and nondominant hand from the Findeis & Weight norms published in Baron (50).

Fine motor dexterity was assessed via the Grooved Pegboard Test (GPT) according to the standardized clinical procedures applied in the collection of the normative data used to compute age-scaled scores (51). Specifically, participants are required to sequentially insert grooved pegs into keyhole shaped holes as quickly as possible using only one hand at a time. Performance was assessed for both dominant and nondominant hand separately, one trial each. The number of pegs dropped was also recorded. An age-scaled score was computed for the completion time to place pegs into all 25 holes for participants over eight years of age. For participants 8 years old and younger, only 10 pegs are administered.

Manual strength of grip (SOG) was measured by use of a hand dynamometer according to standardized procedures employed in deriving the normative data used to compute scaled scores normed according to sex at birth and age (52). This test required the participant to hold and squeeze the dynamometer grip in their hand as tightly as possible. The average strength in kilograms of two trials was recorded for each hand. If the two trials were not within 5 kilograms of one another, a third trial was completed and the average of two trials that were closest to one another was used to compute a norm-referenced score according to age and sex at birth, separately for each hand (52).

Additionally, participants were administered the Beery–Buktenica Developmental Test of Visual–Motor Integration, 6th Edition (Beery VMI; 53). The Beery VMI includes subtexts that isolate skills in visual perception, visual motor integration (VMI), and pencil motor coordinator (VMI-motor). The VMI and VMI-motor subtest age-scaled scores were considered for this study. The VMI subtest requires the participant to copy geometric forms on paper with a pencil. The accuracies of the copied geometric forms are scored with objective scoring criteria outlined in the test manual. The VMI-motor subtest requires the participant draw the same forms within an outline shape that constrains the shape’s form. Age-scaled scored were computed for both of these subtests.

#### 2.3.2 Verbal communication

Assessment of verbal communication was comprehensive in scope, ranging from more basic functions reliant on sensorimotor control of speech, including diadochokinesis and articulation, to more linguistically demanding tasks. Diadochokinesis was quantified as the completion time for 8 repetitions of the consecutive production of the sounds “puh, tuh, kuh,” with better performance being quantified by shorter completion time in seconds, in accordance with standardized procedures outlined in the Oral Speech Mechanism Screening Examination-Third Edition (54). Similarly, the Oromotor Sequences subtest of the NEPSY-II (55), which requires the participant to repeat “tongue twisters” of increasing complexity, is also quantified as a raw score, as age-scaled scores were not available for our age range. For this test, raw scores reflect the total number of items scored as correct, with higher scores indicating better performance. In contrast, articulation errors were totaled as a raw score on the Sounds-In-Words subtest of the Goldman–Fristoe Test of Articulation–Third Edition (GFTA–3; 56), with higher scores indicating more articulation errors. Age-scaled scores were used for tests of rapid naming speed (Inhibition Naming subtest of the NEPSY-II), auditory and visual naming response time (Auditory and Visual Naming Test for Children; AVNT-C; 57), expressive (Expressive Vocabulary Test Third Edition; EVT–3; 58) and receptive vocabulary (Peabody Picture Vocabulary Test™ Fifth Edition; PPVT™–5; 59), and language (CELF–5; 47). For all age-scaled scores, higher scores indicate better performance. Finally, parent report of functional communication and social communication skills were assessed via the age-scaled Functional Communication scale on the Behavior Assessment System for Children-Third Edition (BASC-3; 60) and the total (raw) score on the SCQ (44), respectively. For the BASC-3 Functional Communication score, higher scores indicate better functional communication, whereas, on the SCQ, higher scores indicate greater degree of social communication difficulties.

### 2.4 Data analysis

First, to determine if motor measures demonstrated higher rates of impairment in our ASD group, z tests of independent proportions were computed to compare the proportions of impaired scores (>1.5 SD below the mean of the normative sample) on motor measures between groups. Scores that are >1.5 SD below the normative mean would be labeled as “Below Average” or “Exceptionally Low” according to the American Academy of Clinical Neuropsychology consensus conference statement on uniform labeling of performance test scores (61). Thus, these scored were considered “impaired” for these analyses. One sample Kolmogorov-Smirnov tests for normality indicated that the majority of motor variables were not normally distributed. As such, nonparametric statistical tests were performed to examine group contrasts and correlations. Age-scaled scores for FTT, GPT, SOG, VMI, and VMI Motor were compared between autism and control groups using Mann Whitney U tests. Dominant hand variables resulting in statistically significant group differences were subject to bivariate nonparametric correlations to assess the association between verbal communication and motor skills. Correlations were performed separately for each group. Benjamin–Hochberg procedures were applied to group contrasts and correlation analyses to control for type I error.

### 2.5 Ethics statement

All study procedures were approved by the Institutional Review Board at the University of California-San Francisco (IRB# 11–05249, 21–33613). Parental consent and assent were secured for all participants prior to enrollment.

## 3 Results

**Figure 1:**
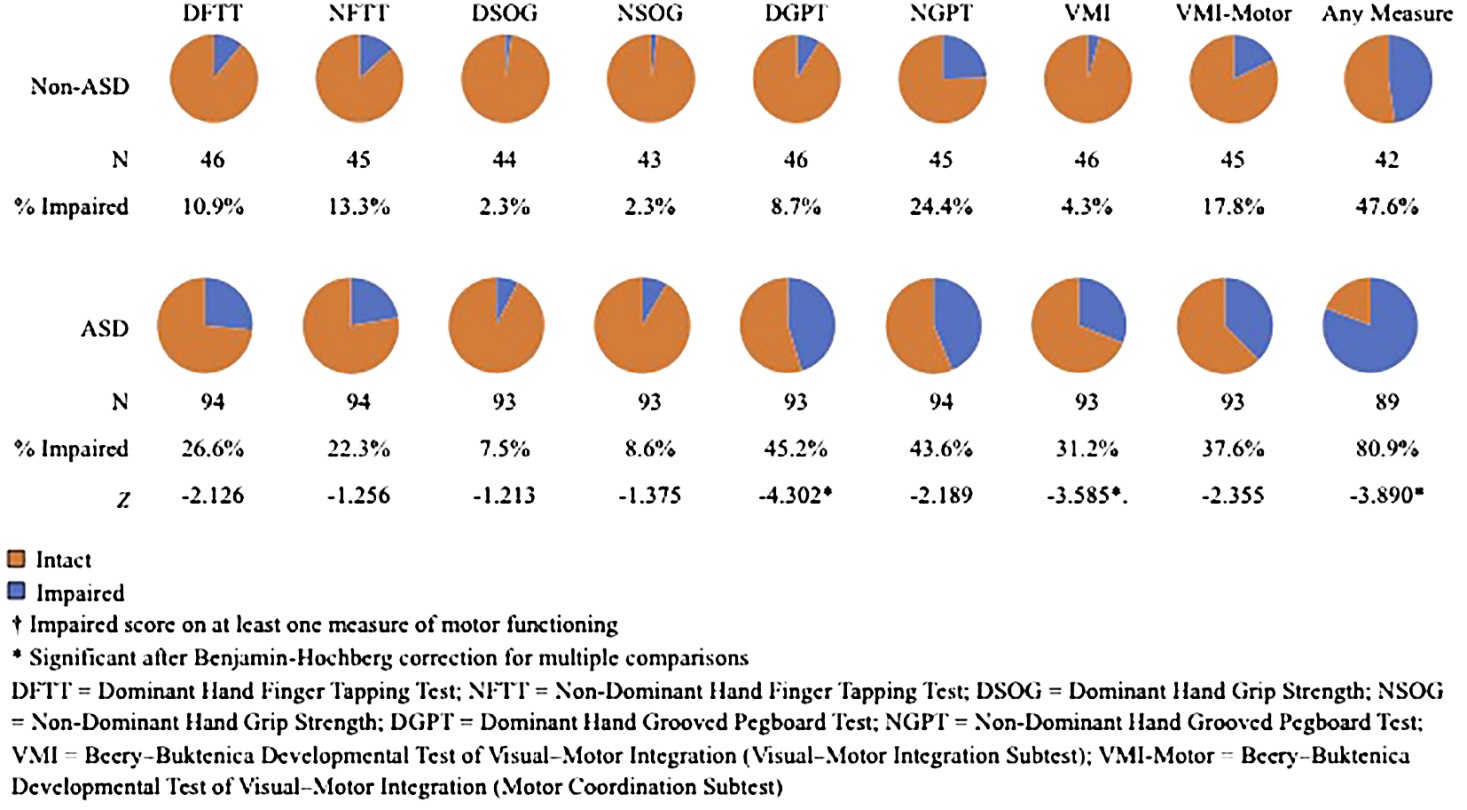
Rates of Impaired Scores on Motor Tasks in the ASD and Non-ASD group.

Impaired scores were significantly more common in the ASD relative to the TDC group for the dominant hand grooved pegboard (z=-4.120, p<.001) and VMI tests (z=3.461, p=.001). The tendency to have at least one impaired score across all motor measures also was significantly greater in the ASD group (z=-3.424, p=.001). These scores indicate higher overall rates of motor impairment in the ASD group. Rates of impaired scores for motor tasks in the ASD and Non-ASD Groups are shown in **Figure 1**.

Results of Mann–Whitney U Tests identified statistically significant group differences in dominant hand finger tapping speed (*U* = 1464.500 p = 0. 002), dominant (*U* = 1011.500, p < 0.001) and non–dominant (*U* =1347.000, p < 0.001) grooved pegboard performance, VMI (*U* = 1250.500, p = 0.001), and VMI motor (*U* = 1350.000, p < 0.001). Significant group differences were not identified for dominant (*U* =1809.500 p = 0.275) and non–dominant (*U* = 1767.000, p = 0.276) grip strength, and non–dominant finger tapping speed (*U* = 1632.500, p = 0.030) after correction for multiple comparisons. Given potential discrepancies between hand preference and performance in autistic individuals (62, 63), we performed *post hoc* group contrasts for right- and left-hand performance on all motor measures. The six participants who described their handedness as ambidextrous were not included in these *post hoc* analyses. Significant group differences were identified for left hand finger tapping speed (U=1431.00, p=.012), and bilaterally for grooved pegboard (U=1005.50, p<.001 for right hand; U=1203.00, p<.001 for left hand) after multiple comparisons correction. Significant group differences were not identified for right hand finger tapping speed (U=1497.50, p=.017), nor for right (U=1711.00, p=.375) or left hand grip strength (U=1636.50, p=.292) after correcting for multiple comparisons.

Although age scaled scores were used for all motor measures, we examined associations via Spearman correlations between the eight age-scaled motor scores and participant age to determine if there was evidence of developmental effects on deviation from the normative mean. After controlling for multiple comparisons, only the ASD group demonstrated a significant correlation between one measure, nondominant hand grip strength, and age (r=-.306, p=.003). Notably, the magnitude of this association was similar for the TDC group; however, it did not achieve statistical significance after controlling for multiple comparisons due to the relatively smaller sample size (r=-.314, p=.041). Given this significant age effect and nonsignificant one sample Kolmogorov-Smirnov test for normality on this variable, a *post-hoc* analysis of covariance was performed to determine whether groups significantly differed in nondominant hand grip strength after controlling for the effects of age. ANCOVA results were nonsignificant (p=.430), indicating that, after controlling for effects of age, groups did not differ in strength of grip.

Spearman correlations between motor skills and verbal communication in the ASD group are presented in **Table 2**. A unique association was identified between slower diadochokinetic rate and slower finger tapping speed, whereas other motor tests had broader associations with a range of communication skills. Specifically, fewer articulation errors and better performance on measures of receptive and expressive language and receptive vocabulary were associated with better fine motor dexterity, pencil motor coordination, and visual-motor integration. Better pencil motor coordination and visual motor integration were additionally associated with better expressive vocabulary, rapid naming, and oromotor sequencing. Finally, pencil motor coordination was the only variable associated with parent report measures of functional and social communication, with better motor coordination being associated with better functional communication skills and fewer social communication symptoms. While only powered to detect large effects, correlation values were generally small for the control group and were not statistically significant after correction for multiple comparisons. These results can be found in the **Supplementary Materials** (**Table 3**).

**Table 2:** Nonparametric correlations (ρ) between measures of motor skills and communication in ASD participants.

Communication Variables (Clinical Measure)	Motor Measures			
	DGPT	VMI	VMI-Motor	DFTT
Diadochokinesis	-0.267	-0.216	-0.223	-0.411*
Articulation (GFTA-3)	-0.402*	-0.381*	-0.388*	-0.232
Oromotor Sequences (NEPSY-II)	0.212	0.446*	0.452*	0.248
Rapid Naming (NEPSY-II)	0.253	0.378*	0.339*	0.162
Auditory Naming Response Time (AVNT-C)	0.341	0.290	0.289	0.021
Visual Naming Response Time (AVNT-C)	0.200	0.249	0.303	0.161
Expressive Vocabulary (EVT-3)	0.238	0.384*	0.472*	0.101
Receptive Vocabulary (PPVT-5)	0.333*	0.397*	0.411*	0.062
Expressive Language (CELF-5)	0.371*	0.521*	0.502*	0.124
Receptive Language (CELF-5)	0.332*	0.475*	0.456*	0.107
Functional Communication (BASC-3)	0.163	0.439*	0.315	0.275
Parent Reported Social Communication (SCQ)	-0.236	-0.367*	-0.291	-0.191

* Significant after Benjamin-Hochberg correction for multiple comparisons.

GFTA-3, Goldman-Fristoe Test of Articulation, Third Edition; EVT-3, Expressive Vocabulary Test, Third Edition; AVNT-C, Auditory and Visual Naming Test - Children; BASC-3, Behavior Assessment System for Children, Third Edition; CELF-5, Clinical Evaluation of Language Fundamentals, Fifth Edition; PPVT-5, Peabody Picture Vocabulary Test, Fifth Edition; SCQ, Social Communication Questionnaire; DGPT, Dominant Hand Grooved Pegboard Test; VMI, Beery–Buktenica Developmental Test of Visual–Motor Integration; VMI-Motor, Beery–Buktenica Developmental Test of Visual–Motor Integration - Motor Coordination; DFTT, Dominant Hand Finger Tapping Test.

**Table 3:** Nonparametric correlations (ρ) between measures of motor skills and communication in non-ASD group.

Communication Variables (Clinical Measure)	Motor Measures			
	DGPT	VMI	VMI-Motor	DFTT
Diadochokinesis	-0.05	-0.047	0.322	-0.326
Articulation (GFTA-3)	0.255	0.061	-0.023	0.252
Oromotor Sequences (NEPSY-II)	0.036	0.157	-0.145	-0.034
Rapid Naming (NEPSY-II)	0.400	0.090	-0.085	0.367
Auditory Naming Response Time (AVNT-C)	0.097	0.045	-0.005	0.048
Visual Naming Response Time (AVNT-C)	0.118	-0.128	-0.063	-0.144
Expressive Vocabulary (EVT-3)	0.180	0.013	0.004	-0.019
Receptive Vocabulary (PPVT-5)	0.211	0.186	0.155	0.069
Expressive Language (CELF-5)	0.056	0.104	0.082	-0.128
Receptive Language (CELF-5)	0.369	0.205	0.281	0.035
Functional Communication (BASC-3)	0.059	0.056	-0.122	0.135
Parent Reported Social Communication (SCQ)	0.002	0.081	-0.132	-0.064

* Significant after Benjamin-Hochberg correction for multiple comparisons.

GFTA-3, Goldman-Fristoe Test of Articulation, Third Edition; EVT-3, Expressive Vocabulary Test, Third Edition; AVNT-C, Auditory and Visual Naming Test - Children; BASC-3, Behavior Assessment System for Children, Third Edition; CELF-5, Clinical Evaluation of Language Fundamentals, Fifth Edition; PPVT-5, Peabody Picture Vocabulary Test, Fifth Edition; SCQ, Social Communication Questionnaire; DGPT, Dominant Hand Grooved Pegboard Test; VMI, Beery–Buktenica Developmental Test of Visual–Motor Integration; VMI-Motor, Beery–Buktenica Developmental Test of Visual–Motor Integration - Motor Coordination; DFTT, Dominant Hand Finger Tapping Test.

## 4 Discussion

The current study compared manual motor abilities of autistic participants to their non–autistic peers in a sample with an age range from late childhood through adolescence. Associations between these motor skills and verbal abilities also were explored. As hypothesized, lower average scores were identified across many motor tests in the ASD group, including dominant hand and left hand finger tapping speed, bilateral manual dexterity, and motor coordination and visual-motor integration on the VMI, whereas no group differences were identified in grip strength. These results are consistent with other research that has demonstrated slower finger tapping in those with higher autistic traits (ages 18–78) (27), difficulty with tasks that require hand–eye–coordination in autistic children and adults (ages 5–33 years) (26) visual–motor integration in autistic children and youth (ages 9–15) (14), and dissociation between hand preference and performance in autistic individuals (62, 63).

When considering the clinical interpretation of this dataset, the majority of participants in the ASD group demonstrated some form of motor impairment (80.9%). Motor impairment was significantly less common in the non-ASD group (47.6%). Notably, the prevalence of motor impairment on any specific measure in the non-ASD group never reached 25%, as low rates of motor impairments were spread across tests. In contrast, the ASD group had high rates of impairment across measures of dexterity, coordination, and visual-motor integration. Rates of impairment in finger tapping speed and grip strength did not significantly differ between groups. Correlations between age and age-scaled scores on motor tests were generally low, suggesting that this is not a developmental effect of impairment in these motor skills. The one exception was that both groups demonstrated a negative correlation of magnitude >.3 between age and nondominant hand age-scaled grip strength scores; however, the age effect did not impact the lack of group differences in strength of grip. Notably, all tests in which higher rates of impaired scores were identified in the ASD group were tasks that required some level of visual-motor integration, whereas finger tapping speed and grip strength tests could technically be performed without looking. This is consistent with prior work describing deficits in sensorimotor integration as a prominent symptom in autism (64).

Consistent with our second hypothesis, we found that poorer performance on tests of motor skills predicted lower verbal communication abilities in autistic participants. First, a unique association was identified for basic motor speed in the manual and motor speech modalities, as slower diadochokinetic rate was associated with slower finger tapping speed. This finding suggests that a slower motor speed generalizes across manual and vocal motor domains of functioning for some individuals with autism.

In contrast, more complex motor tasks requiring dexterity, coordination, and sensorimotor integration were associated with broader range of more complex verbal communication skills. For example, oromotor sequencing, which requires accurate production of tongue twisters, and rapid naming were associated with fine motor coordination and visual-motor integration. These fine motor skills also were strongly associated with articulation and expressive language skills. These findings may reflect a cascade impact of sensorimotor control deficits, including sensorimotor control of speech, on development of speech and language abilities. For example, imbalance in feedforward and feedback control systems has been reported in prior autism studies (3). Although much of this work has focused on visual-motor or postural control, emerging research suggests that auditory-vocal motor control differences are common in autism and related conditions, and are associated with verbal abilities (65, 66).

Differences in motor speech cannot account for all associations, however, as receptive language and vocabulary also were significantly correlated with fine motor coordination and dexterity and visual motor integration. Prior research has demonstrated such associations between motor skills and receptive language abilities in toddlers. For example, Wu et al. (67) found a strong correlation between receptive language abilities and motor functioning in toddlers with ASD. Those with delayed language development had lower motor skills scores than those with typical language development. This suggests a connection between early motor and language impairments in ASD. Findings may support motor-based interventions for language development in young ASD children. In contrast, response time on auditory and visual naming tasks were not significantly associated with any motor skill measures, although after correction for multiple comparisons these analyses were only powered to detect moderate to large effects. Therefore, it is possible that weaker associations exist between fine motor skills and other verbal abilities, such as naming response time, that were not detected in this study.

Finally, pencil motor coordination was the only variable associated with either parent report measure. Specifically, better pencil motor coordination was associated with better functional communication skills and fewer social communication symptoms, suggesting an overall association between core symptoms of autism and fine motor skills. Indeed, Ohara et al. (68) reported a stronger relationship between fine motor skills and social skills than between gross motor skills and social skills. They also noted that object control skills and manual dexterity were the most closely linked to social skills within each motor skill domain. This is consistent with our finding that pencil motor control was significantly associated with functional and social communication skills.

Taken together, these findings suggest that children and adolescents with ASD who have better fine motor skills tend to have better verbal communication skills. This is consistent with previous research showing that motor skills and language development in autistic children are closely linked in early childhood, and may share common neural underpinnings (8, 23). Gowen and Hamilton (69) suggest a dissociation between motor execution and sensory integration for motor planning in autism. In simpler terms, while autistic individuals may not have inherent difficulty executing movements, they might face challenges in processing the sensory information necessary to plan and coordinate those movements effectively. This highlights the importance of examining information processing alongside motor execution when evaluating motor abilities in ASD. Building on this exploration of sensory integration and motor difficulties in autism, we can extend this concept to the realm of communication and language. Similar challenges in processing sensory information might underlie difficulties observed in these areas as well. Just as motor planning may be disrupted by deficits in interpreting sensory cues for movement, so too might the ability to understand and produce speech be affected by problems integrating auditory and visual information or by difficulties tolerating the sensory experience of social interaction (70–75). This highlights the interconnectedness of sensory processing, motor function, communication, and social interaction in the autism spectrum. This collective work suggests that motor control is only one of many factors that may contribute to language development in autistic children. Other factors that were not the focus of the present study, such as genetics, environment, and other cognitive skills, likely also play a role.

Our findings have several implications for the assessment and treatment of autistic children. First, our findings indicate that the majority of ASD participants demonstrated motor impairment on at least one standardized test of manual motor function. This suggests that fine motor skills should be assessed routinely in autistic children, as they may require treatment. Second, our findings of associations between motor and verbal communication abilities suggests that interventions targeting motor skills may also have benefits for development of communication skills. Future research should investigate the causal mechanisms underlying the link between motor control and language abilities in children with ASD.

## Data availability statement

The raw data supporting the conclusions of this article will be made available by the authors, without undue reservation.

## Ethics statement

The studies involving humans were approved by Institutional Review Board at the University of California-San Francisco. The studies were conducted in accordance with the local legislation and institutional requirements. Written informed consent for participation in this study was provided by the participants’ legal guardians/next of kin.

## Author contributions

MS: Conceptualization, Writing – original draft, Writing – review & editing, Formal Analysis, Investigation. GN: Conceptualization, Writing – original draft, Writing – review & editing. VL: Data curation, Formal Analysis, Visualization, Writing – original draft, Writing – review & editing. CD: Conceptualization, Formal Analysis, Funding acquisition, Investigation, Supervision, Writing – original draft, Writing – review & editing, Data curation, Validation.

## References

[1] Iverson JM. Developing language in a developing body: The relationship between motor development and language development. J Child Lang. (2010) 37:229–61. doi: 10.1017/S0305000909990432 PMC2833284 20096145

[2] Karasik LB Tamis-LeMonda CS Adolph KE. Transition from crawling to walking and infants’ Actions with objects and people. Child Dev. (2011) 82:1199–209. doi: 10.1111/j.1467-8624.2011.01595.x PMC3163171 21545581

[3] Bhat AN Landa RJ Galloway JC(. Current perspectives on motor functioning in infants, children, and adults with autism spectrum disorders. Phys Ther. (2011) 91:1116–29. doi: 10.2522/ptj.20100294 21546566

[4] Bhat AN. Is motor impairment in autism spectrum disorder distinct from developmental coordination disorder? A report from the SPARK study. Phys Ther. (2020) 100:633–44. doi: 10.1093/ptj/pzz190 PMC7297441 32154876

[5] Fournier KA Hass CJ Naik SK Lodha N Cauraugh JH. Motor coordination in autism spectrum disorders: A synthesis and meta-analysis. J Autism Dev Disord. (2010) 40:1227–40. doi: 10.1007/s10803-010-0981-3 20195737

[6] Mohd Nordin A Ismail J Kamal Nor N. Motor development in children with autism spectrum disorder. Front Pediatr. (2021) 9:598276. doi: 10.3389/fped.2021.598276 34604128 PMC8480230

[7] Gernsbacher MA Sauer EA Geye HM Schweigert EK Hill Goldsmith H. Infant and toddler oral- and manual-motor skills predict later speech fluency in autism. J Child Psychol Psychiatry Allied Disciplines. (2008) 49:43–50. doi: 10.1111/j.1469-7610.2007.01820.x PMC4123528 17979963

[8] Bedford R Pickles A Lord C. Early gross motor skills predict the subsequent development of language in children with autism spectrum disorder. Autism Res. (2016) 9:993–1001. doi: 10.1002/aur.1587 26692550 PMC5031219

[9] Gernsbacher MA Morson EM Grace EJ. Language and speech in autism. Annu Rev Linguistics. (2016) 2:413–25. 10.1146/annurev-linguist-030514-124824 PMC5260808 28127576

[10] Chen Y Fei X Wu T Li H Xiong N Shen R. The relationship between motor development and social adaptability in autism spectrum disorder. Front Psychiatry. (2022) 13:1044848. doi: 10.3389/fpsyt.2022.1044848 36506435 PMC9726915

[11] Wang LAL Petrulla V Zampella CJ Waller R Schultz RT. Gross motor impairment and its relation to social skills in autism spectrum disorder: A systematic review and two meta-analyses. psychol Bull. (2022) 148:273–300. doi: 10.1037/bul0000358 35511567 PMC9894569

[12] Provost B Lopez BR Heimerl S. A comparison of motor delays in young children: autism spectrum disorder, developmental delay, and developmental concerns. J Autism Dev Disord. (2007) 37:321–8. doi: 10.1007/s10803-006-0170-6 16868847

[13] Liu T Breslin CM. Fine and gross motor performance of the MABC-2 by children with autism spectrum disorder and typically developing children. Res Autism Spectr Disord. (2013) 7:1244–9. doi: 10.1016/j.rasd.2013.07.002

[14] Faber L van den Bos N Houwen S Schoemaker MM Rosenblum S. Motor skills, visual perception, and visual-motor integration in children and youth with Autism Spectrum Disorder. Res Autism Spectr Disord. (2022) 96:101998. doi: 10.1016/j.rasd.2022.101998

[15] Colombo-Dougovito AM Block ME. Fundamental motor skill interventions for children and adolescents on the autism spectrum: A literature review. Rev J Autism Dev Disord. (2019) 6:159–71. doi: 10.1007/s40489-019-00161-2

[16] Zampella CJ Wang LAL Haley M Hutchinson AG de Marchena A. Motor skill differences in autism spectrum disorder: A clinically focused review. Curr Psychiatry Rep. (2021) 23:64. doi: 10.1007/s11920-021-01280-6 34387753

[17] Rinehart NJ Tonge BJ Bradshaw JL Iansek R Enticott PG McGinley J. Gait function in high-functioning autism and Asperger’s disorder: Evidence for basal-ganglia and cerebellar involvement? Eur Child Adolesc Psychiatry. (2006) 15:256–64. doi: 10.1007/s00787-006-0530-y 16554961

[18] Biffi E Costantini C Ceccarelli SB Cesareo A Marzocchi GM Nobile M. Gait pattern and motor performance during discrete gait perturbation in children with autism spectrum disorders. . Front Psychol. (2018) 9:2530. doi: 10.3389/fpsyg.2018.02530 30618953 PMC6297554

[19] Kohen-Raz R Volkman FR Cohen DJ. Postural control in children with autism. J Autism Dev Disord. (1992) 22:419–32. doi: 10.1007/BF01048244 1383190

[20] Esposito G Venuti P. Symmetry in infancy: analysis of motor development in autism spectrum disorders. Symmetry. (2009) 1:215–25. doi: 10.3390/sym1020215

[21] Leezenbaum NB Iverson JM. Trajectories of posture development in infants with and without familial risk for autism spectrum disorder. J Autism Dev Disord. (2019) 49:3257–77. doi: 10.1007/s10803-019-04048-3 31079276

[22] Liu T. Motor milestone development in young children with autism spectrum disorders: An exploratory study. Educ Psychol Pract. (2012) 28:315–26. doi: 10.1080/02667363.2012.684340

[23] Whyatt CP Craig CM. Motor skills in children aged 7–10 years, diagnosed with autism spectrum disorder. J Autism Dev Disord. (2012) 42:1799–809. doi: 10.1007/s10803-011-1421-8 22180003

[24] Chawarska K Paul R Klin A Hannigen S Dichtel LE Volkmar F. Parental recognition of developmental problems in toddlers with autism spectrum disorders. J Autism Dev Disord. (2007) 37:62–72. doi: 10.1007/s10803-006-0330-8 17195921

[25] Morrison S Armitano CN Raffaele CT Deutsch SI Neumann SA Caracci H. Neuromotor and cognitive responses of adults with autism spectrum disorder compared to neurotypical adults. Exp Brain Res. (2018) 236:2321–32. doi: 10.1007/s00221-018-5300-9 29876630

[26] Duffield TC Trontel HG Bigler ED Froehlich A Prigge MB Travers B. Neuropsychological investigation of motor impairments in autism. J Clin Exp Neuropsychol. (2013) 35:867–81. doi: 10.1080/13803395.2013.827156 PMC3907511 23985036

[27] Messing A Apthorp D. Autistic traits are associated with individual differences in finger tapping: An online study. PeerJ. (2023) 11:e15406. doi: 10.7717/peerj.15406 37214091 PMC10198151

[28] Mostofsky SH Powell SK Simmonds DJ Goldberg MC Caffo B Pekar JJ. Decreased connectivity and cerebellar activity in autism during motor task performance. Brain. (2009) 132:2413–25. doi: 10.1093/brain/awp088 PMC2732264 19389870

[29] Schopler E Reichler RJ Renner BR. The childhood autism rating scale (CARS). Los Angeles, CA: Western Psychological Services (2010).

[30] Wilson BN Crawford SG Green D Roberts G Aylott A Kaplan BJ. Psychometric properties of the revised Developmental Coordination Disorder Questionnaire. Phys Occup Ther Pediatr. (2009) 29:182–202. doi: 10.1080/01942630902784761 19401931

[31] Brown T. Movement assessment battery for children: 2nd edition (MABC-2). In: Volkmar FR, editor. Encyclopedia of Autism Spectrum Disorders. New York, NY: Springer (2013). p. 1925–39. doi: 10.1007/978-1-4419-1698-3_1922

[32] Shriver MD Allen KD Mathews JR. Effective assessment of the shared and unique characteristics of children with autism. School Psychol Rev. (1999) 28:538–58. doi: 10.1080/02796015.1999.12085984

[33] Abbott M Bernard P Forge J. Communicating a diagnosis of Autism Spectrum Disorder—A qualitative study of parents’ experiences. Clin Child Psychol Psychiatry. (2013) 18:370–82. doi: 10.1177/1359104512455813 22904114

[34] Gandotra A Kotyuk E Szekely A Kasos K Csirmaz L Cserjesi R. Fundamental movement skills in children with autism spectrum disorder: A systematic review. Res Autism Spectr Disord. (2020) 78:101632. doi: 10.1016/j.rasd.2020.101632

[35] Ghaziuddin M Butler E. Clumsiness in autism and Asperger syndrome: A further report. J Intellect Disabil Res. (1998) 42(1):43–8. doi: 10.1046/j.1365-2788.1998.00065.x 9534114

[36] Berkeley SL Zittel LL Pitney LV Nichols SE. Locomotor and object control skills of children diagnosed with autism. Adapted Phys Activity Q. (2001) 18:405–16. doi: 10.1123/apaq.18.4.405

[37] Libertus K Sheperd KA Ross SW Landa RJ. Limited fine motor and grasping skills in 6-month-old infants at high risk for autism. Child Dev. (2014) 85:2218–31. doi: 10.1111/cdev.12262 PMC4236283 24978128

[38] Mullen EM. Mullen scales of early learning. MN: AGS Circle Pines (1995).

[39] Lloyd M MacDonald M Lord C. Motor skills of toddlers with autism spectrum disorders. Autism : Int J Res Pract. (2013) 17:133–46. doi: 10.1177/1362361311402230 PMC3188325 21610184

[40] Landa R Garrett-Mayer E. Development in infants with autism spectrum disorders: A prospective study: Development in infants with autism spectrum disorders. J Child Psychol Psychiatry. (2006) 47:629–38. doi: 10.1111/j.1469-7610.2006.01531.x 16712640

[41] Deserno MK Fuhrmann D Begeer S Borsboom D Geurts HM Kievit RA. Longitudinal development of language and fine motor skills is correlated, but not coupled, in a childhood atypical cohort. Autism. (2023) 27:133–44. doi: 10.1177/13623613221086448 PMC9806469 35470698

[42] Chukoskie L Townsend J Westerfield M. Motor skill in autism spectrum disorders. In: International Review of Neurobiology, vol. 113. Amsterdam, Netherlands: Elsevier (2013). p. 207–249). doi: 10.1016/B978-0-12-418700-9.00007-1 24290387

[43] American Psychiatric Association. Diagnostic and Statistical Manual of Mental Disorders. Fifth Edition. Virginia, United States: American Psychiatric Association (2013). doi: 10.1176/appi.books.9780890425596

[44] Berument SK Rutter M Lord C Pickles A Bailey A. Autism screening questionnaire: Diagnostic validity. Br J Psychiatry. (1999) 175:444–51. 10.1192/bjp.175.5.444 10789276

[45] Lord C Luyster R Guthrie W Pickles A. Patterns of developmental trajectories in toddlers with autism spectrum disorder. J Consulting Clin Psychol. (2012) 80:477. 10.1037/a0027214 PMC3365612 22506796

[46] Lord C Rutter M Le Couteur A. Autism Diagnostic Interview-Revised: A revised version of a diagnostic interview for caregivers of individuals with possible pervasive developmental disorders. J Autism Dev Disord. (1994) 24(5):659–85. doi: 10.1007/BF02172145 7814313

[47] Wiig EH Secord WA Semel E. Clinical evaluation of language fundamentals: CELF-5. California, United States: Pearson, Thousand Oaks (2013).

[48] Wechsler D. Wechsler intelligence scale for children (2003). Available online at: https://cir.nii.ac.jp/crid/1370567187560684295.

[49] Reitan RM Wolfson D. The halstead-reitan neuropsychological test battery. In: The neuropsychology handbook: Behavioral and clinical perspectives. New York, NY: Springer Publishing Co. (1986). p. 134–60.

[50] Baron IS. Neuropsychological evaluation of the child: Domains, methods, & case studies. Oxford, United Kingdom: Oxford University Press (2018).

[51] Knights RM Norwood JA. Revised smoothed normative data on the Neuropsychological Test Battery for Children. Ottawa, Ontario, Canada: Department of Psychology, Carleton University (1980).

[52] Trites RL. Neuropsychological test manual. Royal Ottawa Hospital, Ottawa Ontario, Canada (1977).

[53] Beery KE Beery NA. The Beery-Buktenica Developmental Test of Visual-motor Integration (Beery VMI): With supplemental developmental tests of visual perception and motor coordination and stepping stones age norms from birth to age six: Administration, scoring, and teaching manual. Pearson, Beery KE, Beery NA. San Antonio, TX: The Beery-Buktenica Developmental Test of Visual-Motor Integration (Beery VMI) (2010).

[54] St. Louis K Ruscello D. Oral Speech Mechanism Screening Examination, 3rd ed. Austin, TX: Pro-Ed (2000).

[55] Korkman M Kirk U Kemp S. NEPSY-II. San Antonio, TX: Pearson (2007). Available at: https://books.google.es/books?id=UukMtwAACAAJ.

[56] Goldman R Fristoe M Pearson Education, I & PsychCorp (Firm). GFTA -3: Goldman Fristoe 3 test of articulation. In: PsychCorp, an imprint of Pearson Clinical Assessment Bloomington. WorldCat, Minnesota (2015).

[57] Hamberger MJ Seidel WT MacAllister WS Smith ML. Auditory and visual naming tests for children. Child Neuropsychol. (2018) 24:903–22. doi: 10.1080/09297049.2017.1414172 PMC6152825 29258379

[58] Williams KT. EVT-3 Expressive Vocabulary Test. 3rd ed. Bloomington, MN: NCS Pearson (2019). Measurement Instrument.

[59] Dunn LM Dunn LM. Peabody Picture Vocabulary Test. 5th ed. Bloomington, MN: NCS Pearson (2019).

[60] Kamphaus RW VanDeventer MC Brueggemann A Barry M. Behavior assessment system for children. In: The clinical assessment of children and adolescents. London, United Kingdom: Routledge (2014). p. 311–26.

[61] Guilmette TJ Sweet JJ Hebben N Koltai D Mahone EM Spiegler BJ. American Academy of Clinical Neuropsychology consensus conference statement on uniform labeling of performance test scores. Clin Neuropsychologist. (2020) 34:437–53. doi: 10.1080/13854046.2020.1722244 32037942

[62] McManus I Murray B Doyle K Baron-Cohen S. Handedness in childhood autism shows a dissociation of skill and preference. Cortex. (1992) 28:373–81. 10.1016/s0010-9452(13)80147-5 1395641

[63] Hauck JA Dewey D. Hand preference and motor functioning in children with autism. J Autism Dev Disord. (2001) 31:265–77. 10.1023/a:1010791118978 11518481

[64] Lidstone DE Mostofsky SH. Moving toward understanding autism: visual-motor integration, imitation, and social skill development. Pediatr Neurol. (2021) 122:98–105. doi: 10.1016/j.pediatrneurol.2021.06.010 34330613 PMC8372541

[65] Russo N Larson C Kraus N. Audio–vocal system regulation in children with autism spectrum disorders. Exp Brain Res. (2008) 188:111–24. 10.1007/s00221-008-1348-2 PMC2774799 18347784

[66] Demopoulos C Kothare H Mizuiri D Henderson-Sabes J Fregeau B Tjernagel J. Abnormal speech motor control in individuals with 16p11. 2 deletions. Sci Rep. (2018) 8:1274. 29352208 10.1038/s41598-018-19751-x PMC5775320

[67] Wu Y-T Tsao C-H Huang H-C Yang T-A Li Y-J. Relationship between motor skills and language abilities in children with autism spectrum disorder. Phys Ther. (2021) 101. doi: 10.1093/ptj/pzab033 33522583

[68] Ohara R Kanejima Y Kitamura M Izawa KP. Association between social skills and motor skills in individuals with autism spectrum disorder: A systematic review. Eur J Invest Health Psychol Educ. (2019) 10:276–96. doi: 10.3390/ejihpe10010022 PMC8314246 34542485

[69] Gowen E Hamilton A. Motor abilities in autism: A review using a computational context. J Autism Dev Disord. (2013) 43:323–44. doi: 10.1007/s10803-012-1574-0 22723127

[70] Minshew NJ Goldstein G. Autism as a disorder of complex information processing. Ment Retard Dev Disabil Res Rev. (1998) 4:129–36. doi: 10.1002/(SICI)1098-2779(1998)4:2<129::AID-MRDD10>3.0.CO;2-X

[71] Haesen B Boets B Wagemans J. A review of behavioural and electrophysiological studies on auditory processing and speech perception in autism spectrum disorders. Res Autism Spectr Disord. (2011) 5:701–14. doi: 10.1016/j.rasd.2010.11.006

[72] Moseley RL Pulvermüller F. What can autism teach us about the role of sensorimotor systems in higher cognition? New clues from studies on language, action semantics, and abstract emotional concept processing. Cortex. (2018) 100:149–90. doi: 10.1016/j.cortex.2017.11.019 29306521

[73] Tryfon A Foster NEV Sharda M Hyde KL. Speech perception in autism spectrum disorder: An activation likelihood estimation meta-analysis. Behav Brain Res. (2018) 338:118–27. doi: 10.1016/j.bbr.2017.10.025 29074403

[74] Hernandez LM Green SA Lawrence KE Inada M Liu J Bookheimer SY. Social attention in autism: neural sensitivity to speech over background noise predicts encoding of social information. Front Psychiatry. (2020) 11:343. doi: 10.3389/fpsyt.2020.00343 32390890 PMC7194032

[75] Key AP D’Ambrose Slaboch K. Speech processing in autism spectrum disorder: an integrative review of auditory neurophysiology findings. J Speech Language Hearing Research : JSLHR. (2021) 64:4192–212. doi: 10.1044/2021_JSLHR-20-00738 PMC9132155 34570613

