# Supplementary material for: Beyond words: an investigation of fine motor skills and the verbal communication spectrum in autism
Source: Front Psychiatry. 2024 May 21;15:1379307. doi: 10.3389/fpsyt.2024.1379307 (PMC11148429; doi:10.3389/fpsyt.2024.1379307)
Supplement: Supplementary file 1 [file Table_1.docx]

Supplementary Material

| *Supplementary Table 1. Nonparametric Correlations (ρ) Between Measures of Motor Skills and Communication in Non-ASD Group* | | | | |
| --- | --- | --- | --- | --- |
|  | Motor Measures | | | |
| Communication Variables (Clinical Measure) | DGPT | VMI | VMI-Motor | DFTT |
|  |  |  |  |  |
| Diadochokinesis | -0.05 | -0.047 | 0.322 | -0.326 |
| Articulation (GFTA-3) | 0.255 | 0.061 | -0.023 | 0.252 |
| Oromotor Sequences (NEPSY-II) | 0.036 | 0.157 | -0.145 | -0.034 |
| Rapid Naming (NEPSY-II) | 0.400 | 0.090 | -0.085 | 0.367 |
| Auditory Naming Response Time (AVNT-C) | 0.097 | 0.045 | -0.005 | 0.048 |
| Visual Naming Response Time (AVNT-C) | 0.118 | -0.128 | -0.063 | -0.144 |
| Expressive Vocabulary (EVT-3) | 0.180 | 0.013 | 0.004 | -0.019 |
| Receptive Vocabulary (PPVT-5) | 0.211 | 0.186 | 0.155 | 0.069 |
| Expressive Language (CELF-5) | 0.056 | 0.104 | 0.082 | -0.128 |
| Receptive Language (CELF-5) | 0.369 | 0.205 | 0.281 | 0.035 |
| Functional Communication (BASC-3) | 0.059 | 0.056 | -0.122 | 0.135 |
| Parent Reported Social Communication (SCQ) | 0.002 | 0.081 | -0.132 | -0.064 |
| * Significant after Benjamin-Hochberg correction for multiple comparisons | | | | |
| GFTA-3 = Goldman-Fristoe Test of Articulation, Third Edition; EVT-3 = Expressive Vocabulary Test, Third Edition; AVNT-C = Auditory and Visual Naming Test - Children; BASC-3 = Behavior Assessment System for Children, Third Edition; CELF-5 = Clinical Evaluation of Language Fundamentals, Fifth Edition; PPVT-5 = Peabody Picture Vocabulary Test, Fifth Edition; SCQ = Social Communication Questionnaire; DGPT = Dominant Hand Grooved Pegboard Test; VMI = Beery–Buktenica Developmental Test of Visual–Motor Integration; VMI-Motor = Beery–Buktenica Developmental Test of Visual–Motor Integration - Motor Coordination; DFTT = Dominant Hand Finger Tapping Test | | | | |
|  |  |  |  |  |
|  |  |  |  |  |
|  |  |  |  |  |
